# “We find what we look for, and we look for what we know”: factors interacting with a mental health training program to influence its expected outcomes in Tunisia

**DOI:** 10.1186/s12889-018-6261-4

**Published:** 2018-12-20

**Authors:** Jessica Spagnolo, François Champagne, Nicole Leduc, Wahid Melki, Myra Piat, Marc Laporta, Nesrine Bram, Imen Guesmi, Fatma Charfi

**Affiliations:** 1 School of Public Health, IRSPUM, Université de Montréal, Montréal, Québec H3N1X9 Canada; 2 School of Public Health, Université de Montréal, Montréal, Québec Canada; 3 Razi Hospital, University of Tunis El-Manar, Tunis, Tunisia; 4 Douglas Mental Health University Institute, McGill University, Montréal, Québec Canada; 5 Montreal WHO-PAHO Collaborating Center for Research and Training in Mental Health, McGill University, Montréal, Québec Canada; 6 Center for School and University Medicine in Manouba, Manouba, Tunisia; 7 Mongi-Slim Hospital, University of Tunis El-Manar, Tunis, Tunisia

**Keywords:** Implementation, *mhGAP*, Training, Mental health, Primary care, Physicians, Case study, Tunisia

## Abstract

**Background:**

Primary care physicians (PCPs) working in mental health care in Tunisia often lack knowledge and skills needed to adequately address mental health-related issues. To address these lacunas, a training based on the *Mental Health Gap Action Programme (mhGAP) Intervention Guide (IG)* was offered to PCPs working in the Greater Tunis area between February and April 2016. While the *mhGAP-IG* has been used extensively in low- and middle-income countries (LMICs) to help build non-specialists’ mental health capacity, little research has focused on how contextual factors interact with the implemented training program to influence its expected outcomes. This paper’s objective is to fill that lack.

**Methods:**

We conducted a case study with a purposeful sample of 18 trained PCPs. Data was collected by semi-structured interviews between March and April 2016. Qualitative data was analyzed using thematic analysis.

**Results:**

Participants identified more barriers than facilitators when describing contextual factors influencing the *mhGAP-*based training’s expected outcomes. Barriers were regrouped into five categories: structural factors (e.g., policies, social context, local workforce development, and physical aspects of the environment), organizational factors (e.g., logistical issues for the provision of care and collaboration within and across healthcare organizations), provider factors (e.g., previous mental health experience and personal characteristics), patient factors (e.g., beliefs about the health system and healthcare professionals, and motivation to seek care), and innovation factors (e.g., training characteristics). These contextual factors interacted with the implemented training to influence knowledge about pharmacological treatments and symptoms of mental illness, confidence in providing treatment, negative beliefs about certain mental health conditions, and the understanding of the role of PCPs in mental health care delivery. In addition, post-training, participants still felt uncomfortable with certain aspects of treatment and the management of some mental health conditions.

**Conclusions:**

Findings highlight the complexity of implementing a *mhGAP*-based training given its interaction with contextual factors to influence the attainment of expected outcomes. Results may be used to tailor structural, organizational, provider, patient, and innovation factors prior to future implementations of the *mhGAP-*based training in Tunisia. Findings may also be used by decision-makers interested in implementing the *mhGAP-IG* training in other LMICs.

**Electronic supplementary material:**

The online version of this article (10.1186/s12889-018-6261-4) contains supplementary material, which is available to authorized users.

## Background

Authors have strongly advocated for further integrating mental health in primary care settings [1–5] to address the mental health treatment gap, which is especially alarming in low- and middle-income countries (LMICs) [3, 6–9]. A plethora of factors cause this gap, including, but not limited to, insufficient and unevenly distributed mental health resources [10–14]. For example, out of the limited number of health workers with mental health competencies and skills, the majority work in high-income countries (HICs) [10, 13, 15, 16], despite an estimated three-quarters of the global disease burden caused by such disorders affecting LMICs [17]. Untreated mental health issues are associated with increased mortality and disability rates, reducing the life expectancy of people living with serious mental disorders by up to 20 years on average [18–20].

A strategy encouraged by the *World Health Organization* (*WHO*) to tackle the limited number and unequal distribution of mental health workers is the use of non-specialists [21, 22]. To prepare them for their role in mental health care, and to scale up such services, trainings based on the *Mental Health Gap Action Programme* (*mhGAP*) *Intervention Guide* (*IG*), which regroups evidence-based interventions for what the *WHO* considers priority conditions [23–25], have been encouraged. These priority conditions include depression, psychosis, bipolar disorder, epilepsy, developmental and behavioural disorders, dementia, alcohol and drug use disorders, and suicide/self-harm [23, 25]. The *mhGAP-IG (version 1.0)* was first launched in 2010 [23], and has since been updated to *version 2.0* based on new evidence and extensive feedback from those who used the first version [25]. While the *mhGAP*-based training, in both of its versions, has been implemented in over 100 countries [26, 27], little research has focused on how factors within specific contexts interact with the implemented training program to influence its expected outcomes [27–31]. Such findings highlight real-world challenges to the training’s uptake and scale-up in specific resource-limited settings [32–34] and may encourage decision-makers to create a system facilitating non-specialists’ involvement in mental health care [4, 27, 35–37].

We developed an exploratory trial [38, 39] that seeks to contextualize, implement, and evaluate a mental health training program for primary care physicians (PCPs) in the Greater Tunis area of Tunisia based on the *mhGAP-IG (version 1.0)* [23] before country-wide implementation. The trial has two objectives. First, with a randomized controlled trial, we aimed to assess the potential value of capacity building by training PCPs working in primary care settings in the Greater Tunis area using a training based on the *mhGAP-IG (version 1.0)* [23]. We hypothesized the training would improve PCPs’ mental health knowledge, attitudes, perceived self-efficacy, and self-reported practice. Results will be published in a separate paper. The second objective, the results of which are presented in this paper, was to identify contextual factors that interacted with the implemented training to influence its expected outcomes. This evaluation type is referred to as Type III implementation analysis [33, 40], a current priority in global mental health [15].

To our knowledge, this is the first documentation of such factors after the implementation of a mental health training program in Tunisia. Our findings will help build research capacity in Tunisia [41] and in LMICs more generally [15, 42]. Our findings will also add to the limited (but growing) peer-reviewed research on the *mhGAP-IG* training [27], all the while highlighting crucial information to prepare for the program’s country-wide use in Tunisia [43].

### Implementing a training based on the *mhGAP-IG* in Tunisia

Tunisia, a lower-middle income North African country [44], is among the many nations worldwide making mental health a priority [4, 45], particularly because of the recorded rise of mental health problems, substance use disorders, and suicide rates since the 2010–2011 Revolution, which protested high levels of youth unemployment, political repression, and government corruption [41, 46–52]. The development and adoption of the *2013 Tunisian Mental Health Strategy* aims to facilitate the transition from institutional to community-based mental health care. This transition strives to expand access to needed mental health services [41], notably through the revival of continuing mental health education programs [41, 43]. While mental health training programs have been offered to PCPs in the past, these were implemented under the leadership of individual governorate directors, and not under a national program. Thus, training implementation was previously conducted non-systematically. In addition, these training programs were general and thematic lectures about mental health and illness, with limited interactive components and mental health resources for trainees.

A training based on an adapted version of the *mhGAP-IG (version 1.0)* [23] was implemented as a pilot initiative between February and April 2016. Collaborators include the Presidents of the *Committee for Mental Health Promotion* and *Technical Committee Against Suicide* at the level of the Ministry of Health in Tunisia, the *School of Public Health* at *Université de Montréal* (Québec, Canada), the *WHO* office in Tunisia, and the Montreal *WHO-Pan American Health Organization (PAHO) Collaborating Center for Research and Training in Mental Health* (Québec, Canada). The training’s goal was to increase PCPs’ mental health competencies and skills [41, 53, 54], thus further encouraging mental health’s integration in primary settings, increasing access to effective services, and creating proximity mental health services [41, 43, 55].

Training details have been described elsewhere [56]. In brief, *mhGAP-IG (version 1.0)* modules [23] were selected by members of the Tunisian Ministry of Health and adapted to meet the primary care realities of the Greater Tunis area. Training included modules on depression, psychosis, self-harm/suicide, and alcohol/drug use disorders, chosen to meet the country’s pressing mental health needs. First, data suggests that consultations specifically for anxiety and depression have increased after the Tunisian Revolution [41, 46, 47]. Second, records show that the number of deaths by suicide rose approximately two times and self-immolation, three times during the 4 years following the Revolution [50, 51]. Third, rates of substance use (specifically of opioids, cannabis, ecstasy, and alcohol) and substance use disorders have reportedly increased, especially among those under 35 years of age [41, 48]. Last, in Tunisia, it is reported that annual mortality rates associated with schizophrenia have increased given its link with deaths by suicide [52]. A general introduction to the *mhGAP*, the *IG*, and the module “*General Principles of Care*” were also included in the training. Training sessions were facilitated by Tunisian psychiatrists and supported by PCPs working to promote continuing mental health training in the Greater Tunis area (i.e., tutors), all trained in the proper use of the *mhGAP-IG*. Training sessions, offered once a week for 5 weeks, included general lectures, role plays, and group discussions. These were followed by a support session where trainer-psychiatrists facilitated clinical case discussions and role plays. In total, the training program lasted 6-weeks.

### Objective of the paper

With the present paper, we aim to identify contextual factors that interacted with the implemented mental health training program based on the *mhGAP-IG (version 1.0)* to influence its expected outcomes in the Greater Tunis area of Tunisia.

## Methods

### Conceptual framework

We chose Chaudoir and colleagues’ (2013) framework [57] to guide this paper because it builds upon two pre-existing and widely used frameworks [32, 58] by adding patient factors to their unifying four-factor constructs. Exploring patient factors is particularly important to our paper, since mental illness’s stigma may prevent patients from seeking professional help, which has been shown to perpetuate the mental health treatment gap [37, 59].

Chaudoir and colleagues’ (2013) framework [57] consists of the following categories: 1) structural factors (i.e., the outer setting comprising the broader sociocultural context or community); 2) organizational factors (i.e., characteristics of the organization where providers use the intervention); 3) provider factors (i.e., characteristics of those implementing the intervention); 4) innovation factors (i.e., characteristics of the implemented intervention); and 5) patient factors (i.e., characteristics of those receiving the intervention from providers).

**Fig. 1:**
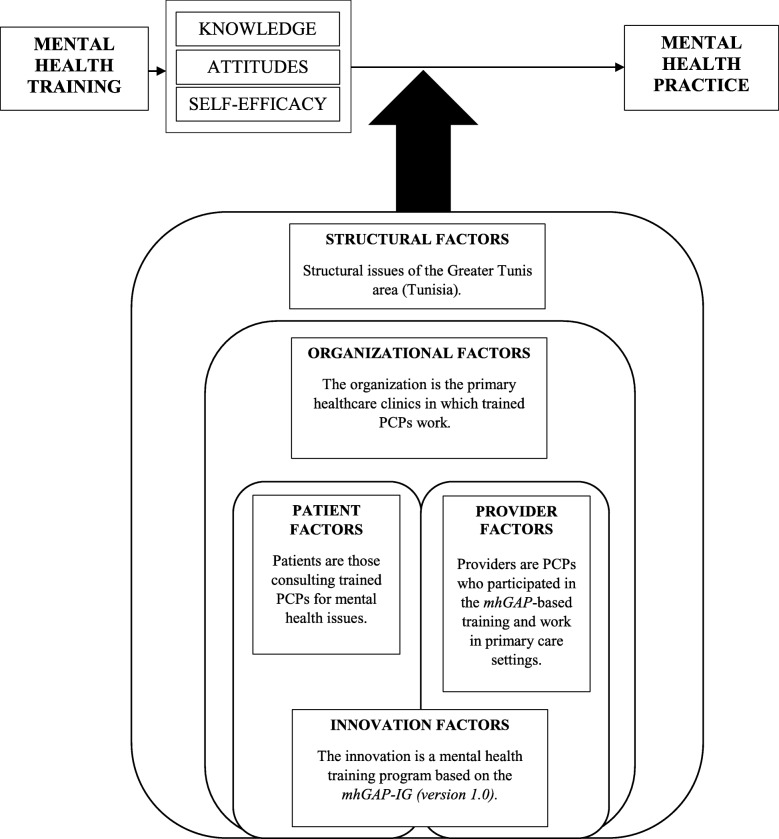
Conceptual framework. Illustrates our multi-factor framework, and is based on the one developed by Chaudoir and colleagues [57]. It highlights the following categories that interacted with the implemented training program to influence its expected outcomes: 1) structural factors (i.e., the outer setting comprising the broader sociocultural context or community); 2) organizational factors (i.e., characteristics of the organization where providers use the intervention); 3) provider factors (i.e., characteristics of those implementing the intervention); 4) innovation factors (i.e., characteristics of the implemented intervention); and 5) patient factors (i.e., characteristics of those receiving the intervention). For this paper’s purposes, our conceptual framework was used to develop interview questions, as well as to analyze and sort data

Figure 1 illustrates our multi-factor framework. For this paper’s purposes, it was used to develop interview questions, as well as to analyze and sort data.

### Study design

We conducted a *case design with three embedded levels of analysis* [60, 61], the case being the organization of a mental health training program based on the *mhGAP-IG*, offered to PCPs working in the Greater Tunis area. Three factors influenced this design. Firstly, the case study method is suggested when conducting Type III implementation analysis [33]. Secondly, the *single case design* was chosen because our case is a *common case* [60]. More specifically, the Greater Tunis area is often where interventions are piloted, given the setting’s diversity (i.e., urban, rural, semi-urban, and semi-rural), which is representative of other areas of Tunisia. Therefore, lessons learned from the in-depth exploration of factors perceived to interact with the implemented training to prevent the attainment of its expected outcomes may help shed light on such factors in other areas of Tunisia [60, 62]. Lastly, the case study has *embedded levels of analysis* [60] because our aim was to identify contextual factors interacting with the implemented training to influence its expected outcomes according to a multi-factor framework [57]. While Chaudoir and colleagues (2013) [57] identify five levels in their framework, these may be regrouped into three levels of explanation [60]: structural (i.e., the health system in the Greater Tunis area), organizational (i.e., primary healthcare clinics’ organizational context), and individual (i.e., provider, patient, and innovation factors, (i.e., provider, patient, and innovation factors).

### Study settings and participants

We conducted the exploratory trial in the four governorates of the Greater Tunis area: Ariana, Tunis, Ben Arous, and Manouba. Sampling for the larger trial in which this paper is inscribed has been described in detail elsewhere [54]. In brief, a total of 112 PCPs were randomized to either Group 1 or Group 2. Both groups received the training, but at different times. Specifically, Group 1 received the training between February and March 2016, whereas Group 2 received the training between March and April 2016. Forty-five PCPs in Group 1 completed the training program. To recruit participants for this paper, the first author contacted by telephone the 45 PCPs who had completed the first round of training offered in February–March 2016. Since these PCPs already met eligibility criteria for the exploratory trial [54] and had an in-depth understanding of the *mhGAP*-based training, the sampling method was purposeful [61]. Of the 45 PCPs contacted, 27 agreed to be interviewed. Nine PCPs decided not to participate in the interviews after initial agreement, given other commitments, which resulted in interviews with 18 participants.

Questionnaires designed for the exploratory trial were administered prior to randomizing participants to either Group 1 or Group 2. Therefore, we had the socio-demographic and practice characteristics of the 18 PCPs who agreed to participate in the interviews. This descriptive data is presented in Table 1.

**Table 1:** Characteristics of the PCPs in the study prior to the implementation of the training (*n* = 18)

Characteristics	Continuous variables	Categorical variables
Socio-demographic characteristics	M (SD) (Q1, Q2, Q3)	n (%)
Age (in years)	47.8 (4.2) (44.8, 48.0, 52.3)	–
Women	–	16 (88.9)
Born in Tunisia	–	18 (100)
Mother tongue, Arabic	–	18 (100)
Medical school in Tunisia	–	16 (88.9)
Practice characteristics	M (SD) (Q1, Q2, Q3)	n (%)
Governorate		
Ariana	–	6 (33.3)
Tunis	–	5 (27.8)
Ben Arous	–	4 (22.2)
Manouba	–	3 (16.7)
Mental health training in the last 12 months (yes)	–	4 (22.2)
Average number of years working as a PCP	18.2 (5.3) (12.8, 18.0, 21.5)	–
Hours work / week ^a^	35.5 (3.2) (36.0, 36.0, 36.0)	–
Average number of patient consultations / week	138.1 (45.1) (100.0, 120.0, 180.0)	–
Average number of consultations for mental health / week	17.0 (12.7) (8.3, 15.3, 21.9)	–
Average number of consultations for mental health / week ^a^	–	–
By appointment	2.4 (3.9) (0.0, 1.0, 2.6)	
Without appointment	14.5 (13.3) (6.2, 12.5, 18.6)	
Average number of hours dedicated to mental health care / week ^a^	4.2 (2.5) (2.3, 3.6, 6.2)	–
% of mental health consultations per week according to diagnosis: Types of mental health consultation per week:		
Anxiety	53.0 (28.3) (30.0, 50.0, 82.5)	–
Depression	33.7 (23.1) (22.3, 30.0, 42.5)	–
Alcohol use disorders	6.2 (7.6) (0.0, 5.0, 10.0)	–
Psychosis (including schizophrenia)	5.2 (5.8) (0.8, 2.5, 10.0)	–
Drug use disorders	3.9 (4.1) (0.0, 2.5, 8.5)	–
Self-harm/ suicide	1.8 (2.2) (0.0, 1.0, 2.3)	–
% of mental health clientele mean		
Referred to specialized care ^a^	59.6 (32.0) (50.0, 60.0, 85.0)	–
Receiving support (ex.: active listening)	50.7 (33.9) (30.3, 50.0, 80.0)	–
Receiving psychoeducation	43.6 (35.1) (7.5, 50.0, 80.0)	–
Receiving pharmacology	42.7 (37.6) (1.8, 40.0, 82.5)	–
Receiving psychotherapy	10.6 (18.3) (0.0, 0.0, 15.0)	–
Average number of follow-up visit/ patient with mental health issues	4.7 (2.2) (3.0, 4.0, 5.3)	–

^a^Missing values were greater than 5% but less than 10%

### Data collection

For this paper, data was collected in March and April 2016 by semi-structured individual and group interviews. Four were group interviews, with PCPs from the governorate of Ariana (*n* = 6), Manouba (*n* = 2), Ben Arous (*n* = 4), and Tunis (*n* = 3).^1^ Three PCPs participated in individual interviews because they could not attend the scheduled group interviews. These included one PCP from Manouba, and two PCPs from Tunis. Group interviews lasted between 70 and 90 min and individual interviews between 50 and 70 min. All interviews were conducted in French by the first author. In Tunisia, French is the language in which medical school is taught, and all medical staff is fluent.

An interview guide with open-ended questions based on the framework developed by Chaudoir and colleagues [57] was developed by the first author and her doctoral supervisors (FC and NL) (see “Additional file 1”). Questions match Chaudoir and colleagues’ [57] five categories and cover: 1) structural issues affecting mental health care by PCPs in the Greater Tunis area, such as mental health policies, social context, local workforce, and aspects of the physical environment; 2) organizational factors affecting the ways in which mental health care is delivered by PCPs and supported within primary healthcare clinics; 3) provider factors, such as specific characteristics that might influence PCPs’ use of the mental health training and involvement in the field of mental health; 4) innovation factors, such as PCPs’ perceptions of the training (i.e., its compatibility with primary care context and its quality); and 5) patient factors, such as patients’ characteristics that might influence health-related beliefs. Individual and group interviews were audio recorded and transcribed verbatim.

### Data analysis

Qualitative data analyses were conducted using deductive and inductive approaches [61] and necessitated multiple steps. First, the interview guide developed from Chaudoir and colleagues’ (2013) categorical framework [57] served as a “template” for coding [61, 63, 64] and was used to develop a preliminary code book before the coding process began [61, 64, 65]. Second, all transcripts were checked and read thoroughly by the first author before coding, which allowed for a general understanding of the data. Third, four initial transcripts were coded by the first author using the preliminary code book. During this phase, new codes that emerged were added to the code book [65]. Fourth, the first author proceeded to regroup codes into sub-themes and themes, which were compared to Chaudoir and colleagues’ (2013) categories [57]. Codes that did not fit into Chaudoir and colleagues’ (2013) framework [57] include PCPs’ descriptions of the training’s impact on their competencies and practice, as well as suggested recommendations to improve the training program and mental health care delivery in the Greater Tunis area. PCPs’ competencies and skills were regrouped into “positive” or “negative” effects, and codes associated with these effects were counted [65]. Sub-themes regrouped into Chaudoir and colleagues’ (2013) framework [57] were divided into two categories: facilitators and barriers. Codes associated with “facilitators” and “barriers” were counted [65]. Fifth, the first author presented the preliminary code book and regrouped codes with accompanying illustrative examples and citations to her doctoral supervisors for approval. During this phase, codes, sub-themes, and themes were discussed. New codes, sub-themes, and themes were generated, specifically related to providers’ descriptions of the training’s impact on their competencies and skills, and provider factors inscribed within Chaudoir and colleagues’ (2013) framework [57]. Once agreement on codes, sub-themes, and themes was obtained between the first author and her doctoral supervisors, the first author coded the remaining transcripts. An overview of the codes (and their categorization into positive/negative effects or facilitators/barriers, where applicable), sub-themes, and themes included in the final code book is presented in “Additional file 2.”

Socio-demographic and practice characteristics of the 18 participating PCPs were analyzed using SPSS version 25.0 [66], and descriptive statistics were reported. Group frequencies and percentages were reported for categorical variables. Means, standard deviations (SD), as well as quartiles 1 (Q1), 2 (Q2 – the median), and 3 (Q3) were reported for continuous variables.

### Scientific rigor

Validity checks are recommended when conducting qualitative research [65]. We employed member-checking, multiple data examiners, and triangulation of multiple data sources [61, 65]. Member-checking entails taking a findings summary back to the participants who provided the original data and asking them if the data reflects their reality [65]. The first author, her doctoral supervisors, the *WHO* office in Tunisia, and the Presidents of the *Committee for Mental Health Promotion* and *Technical Committee Against Suicide* organized a dissemination session in Tunis on 22 September 2017, where preliminary research findings from the exploratory trial were shared, including preliminary codes, sub-themes, themes, and supporting examples. The Presidents of the *Committee for Mental Health Promotion* and *Technical Committee Against Suicide* invited all 112 PCPs of the larger trial (which included PCPs who participated in individual or group interviews for this paper), trainer-psychiatrists, PCPs responsible for continuing medical education in the Greater Tunis area, and governorate directors. In total, 61 participants were present at the dissemination session, including the Presidents of the *Committee for Mental Health Promotion* and *Technical Committee Against Suicide*. This session helped validate preliminary findings and generate discussions around their key themes, which in turn became the basis for recommendations on ways to ensure effective mental health care delivery in primary care settings. These recommendations, drafted in collaboration with the different stakeholder groups present at the session, were the basis of a report written by the first author and validated by the Presidents of the *Committee for Mental Health Promotion* and *Technical Committee Against Suicide* before being sent to all session attendees.

A second validity strategy employed was the inclusion of multiple data examiners. The preliminary code book developed by the first author was presented to her two doctoral supervisors for feedback. The supervisors provided feedback on the codes, sub-themes, themes, and data associated with the four initial transcripts coded [65]. This process ensured accuracy of data analysis and data reporting.

The last validity strategy employed was the triangulation of multiple data sources, which took two different forms in the trial. First, by interviewing PCPs from different governorates of the Greater Tunis area of Tunisia and with diverse experiences in mental health, we were able to check for the consistency of what was shared about the same issue [61]. Second, Patton [61] suggests no single method is ever adequate to reveal a research problem’s different facets. Therefore, the qualitative findings presented in this paper will be used to complement results of the randomized controlled trial. This complementarity enabled us to generate findings contributing to our overall understanding of a *mhGAP*-based training’s impact on its expected outcomes [61].

## Results

Results are presented in three parts. The first part describes participants’ perceptions of the training’s impact on their competencies and practice (i.e., expected outcomes). Codes are regrouped into two main categories: positive effects (15 codes) and negative effects (5 codes). The second part highlights contextual factors interacting with the implemented training to influence its expected outcomes. Codes are regrouped under five factors [57], which are divided into key themes and sub-themes. Codes are then regrouped into two main categories: barriers (37 codes) and facilitators (31 codes). The third part explores participants’ recommendations to address these barriers, specifically by improving the training program and the ways PCPs deliver mental health care in the Greater Tunis area.

### Part 1: PCPs’ perceptions of the training’s impact on their competencies and practice

After participation in the training, PCPs shared the program’s mostly positive effects on their competencies and practice. Most PCPs appreciated their increased familiarity with pharmacological treatments. After the training, they were better able to decide whether to prescribe medication to patients presenting with mental health issues and to identify which medications should be prescribed. For example, the training taught them that antidepressants may be considered for moderate-severe depression, but less so for minor depression. This new knowledge increased PCPs’ confidence to prescribe, change patients’ medications, or renew existing prescriptions. Post-training, PCPs felt more knowledgeable about symptoms related to mental illness, which increased their confidence in treating patients. For example, new knowledge among trainees commonly included being able to ask patients about suicidal thoughts without worrying they might increase their suicide risk.

Most PCPs mentioned improvements in attitudes towards mental health and illness. According to them, the training helped demystify certain beliefs about mental health issues and mental health care in non-specialized settings. For example, after the training, most PCPs acknowledged substance use disorders as illnesses, not moral, personal faults. This change in perception allowed PCPs to understand that many people living with substance use disorders suffer in silence and it encouraged them to view people presenting with such disorders in the way they would patients consulting for physical conditions. In addition, after training, most PCPs understood that not all mental health issues require specialized care:

*“Before I thought all these [mental health] pathologies should be referred to psychiatrists, psychologists, child psychiatrists, or others. The training helped me demystify things and made me take care of those patients.” (Interview 2, participant 5)*

With this new understanding, PCPs’ interest and investment in mental health increased. Hence, post-training, they wanted to allocate additional time to people consulting for mental health issues and ensure adequate follow-up. For example, since many patients with mental health issues come to the clinic solely to pick up medication every 15 days, PCPs would make it a point to check in with them.

Post-training, PCPs shared that they more comfortably engaged with patients to obtain information that could help them pose a mental health diagnosis. Specifically, most PCPs learned how to guide their interrogation (for example, by asking “good” questions suggested during the training) when mental health problems were suspected among patients. Knowing how to detect symptoms related to mental illness and to ask these “good” questions encouraged PCPs to be more aware of mental health conditions in practice, regardless of patients’ consultation motives:

*“The pathology of mental illness is frequent [in our area]. But, we find what we look for, and we look for what we know […] now we uncover a lot more, especially cases of depression.” (Interview 5, participant 13)*

Post-training, PCPs learned how to expand their treatment repertoire beyond pharmacology. PCPs were more inclined to consider psychosocial interventions. Greater confidence in prescribing medications and engaging in psychosocial interventions has, according to PCPs, increased the number of patients they treat for mental health issues weekly. In addition, they are more inclined to ensure greater continuity of care:

*“For all patients with schizophrenia, I informed the nurses to remind me to see them at least every 3 months. It is necessary to keep a contact between the patient and the doctor.” (Interview 3, participant 7)*

Not all PCPs thought the training improved their mental health competencies and skills. While most PCPs did acknowledge an increase in their knowledge about medication, some said they were still unfamiliar with certain aspects of pharmacology. Despite training, PCPs still did not possess enough knowledge about medications’ side effects, interactions among molecules, or suggested treatment length, often preventing PCPs from having the courage to prescribe certain medication types (ex.: neuroleptics and antipsychotics). Some PCPs also shared that while the training helped demystify the field of mental health, they still feared treating schizophrenia, psychosis, and substance use disorders given perceived limited capabilities. While they can recognize these disorders in practice, they still believe these illnesses always necessitate treatment and follow-up in specialized care.

### Part 2: Exploring contextual factors that influenced the implemented training’s expected outcomes

Results show that contextual factors interacted with the implemented training to influence its expected outcomes illustrated in Part 1. The subsequent sections present these contextual factors, organized according to Chaudoir and colleagues’ framework [57], and how they facilitated or challenged PCPs’ competencies and skills (also presented in Table 2).

**Table 2:** Barriers and facilitators influencing the implemented training’s expected outcomes

Dimension	Barriers	Facilitators
Structural factors	• PCPs cannot prescribe certain molecules. • Substance use disorders are often managed judicially. • PCPs feel that physical health is valued more than mental health. • Mental health statistics are not taken seriously. • PCPs still use “ancient” mental health tools in practice. • Substance use disorders are stigmatized in Tunisia. • Mental health care within institutions is stigmatized. • There is a lack of continuity in mental health trainings. • There is a lack of obligatory mental health internships after medical school to further develop professional practice. • If there are mental health trainings, not all PCPs can attend. • There is only one mental health hospital in the country, and it is not accessible to all.	• Laws and restrictions are changing to reflect current trends in mental health. • There is increased attention put on mental health statistics. • Mental health is recognized in the country through the development of the national programme for mental health promotion. • Strategies are used to increase awareness of mental health conditions. • There is less stigma towards certain types of mental disorders since the Revolution. • The Ministry adopted a new medical curriculum, encouraging increased teachings and internships in mental health for future family physicians.
Total	11 barriers	6 facilitators
Organizational factors	• Trained PCPs are not always at the same primary healthcare clinic, affecting continuity in care. • There is a lack of medication in primary healthcare clinics. • If medication is available, it is easily stolen. • If medication is available, it is not evenly distributed. • If medication is available, it runs out quickly. • There is a lack of time to provide adequate mental health care. • There is a high turnover of employees within primary healthcare organizations. • PCPs expressed difficulties working with other health care professionals in the primary healthcare clinic. • Primary healthcare clinics do not encourage staff meetings. • Collaborations with the mental health hospital is difficult.	• Medication is available within primary healthcare clinics. • PCPs engage in case discussions with colleagues about mental health. • Collaborations with PCPs responsible for continuing medication education helps with mental health care. • There are opportunities for collaborations with other healthcare professionals.
Total	10 barriers	4 facilitators
Provider factors	• PCPs do not have previous mental health training. • PCPs do not like treating certain types of mental illnesses. • PCPs do not get involved with pharmacological treatment. • PCPs are not interested in mental health.	• PCPs have participated in mental health trainings. • PCPs participated in a mental health internship during medical school. • Many years of field experience have equipped PCPs with confidence in their general clinical skills. • PCPs are personally motivated to provide mental health care. • PCPs have personal preferences for certain types of illnesses. • PCPs participate in mental health training during their own time (outside of office hours).
Total	4 barriers	6 facilitators
Patient factors	• Patients think that receiving care in primary healthcare clinics is sub-par to receiving care by a specialist. • Patients are treated differently once “society” knows they live with mental health issues. • Patients do not seek care because they are afraid of legal issues. • Patients do not seek care because they do not want to be noticed by community members. • In consultation, patients are interrupted by other patients. • Patients are not aware that mental health services are available at primary healthcare clinics. • Patients do not know that mental health services are confidential.	• Patients prefer seeking and receiving care at the primary healthcare clinic because it is less stigmatizing than the mental health hospital. • Patients like receiving care at the primary healthcare clinic because they may go unnoticed. • Patients like receiving care at the primary healthcare clinic because it is offered quickly. • Patients think that the mental health hospital is very stigmatizing. • Patients think that the mental health hospital is too far. • Patients think that receiving services at the mental health hospital takes too long. • Patients are more open about their own mental health. • Patients will seek care at the primary healthcare clinic between appointments with psychiatrists.
Total	7 barriers	8 facilitators
Innovation factors	• Modules chosen do not correspond to the clientele seen by PCPs. • PCPs did not like all the theory provided during the training. • PCPs did not like that they were not able to learn about all the modules included in the guide. • PCPs did not like role plays. • PCPs found there was not enough time for all the content provided.	• Modules chosen correspond to the reality seen by PCPs. • Modules chosen correspond to the reality of the Greater Tunis area. • PCPs appreciated the clinical discussions during the training as they helped orient practice. • PCPs liked the role plays because they helped learning. • PCPs liked that they could learn from their peers. • PCPs enjoyed the videos shown during the training. • PCPs liked the training guide.
Total	5 barriers	7 facilitators
TOTAL	37 barriers	31 facilitators

#### Structural factors

PCPs highlighted more barriers (11 codes) than facilitators (6 codes) when describing broader context or community factors interacting with the implemented training to influence its expected outcomes.

##### Public policies

PCPs explained that restrictions challenge their involvement in pharmacological treatment, especially when prescribing *Haloperidol (*e.g.*, Haldol)* and *Lorazepam (*e.g.*, Temesta)*, two listed medications in the *mhGAP-IG (version 1.0).* Thus, while these medications are available in Tunisia, these restrictions make PCPs believe that only psychiatrists can prescribe them.^2^ In addition, PCPs stated that substance use disorders are often criminalized. For example, there are criminal sanctions for minor drug consumption and possession for personal use. These judicial implications, according to participants, restrict their involvement in care because they fear legal repercussions for their patients. However, PCPs were optimistic about certain changes in legislation. Revisions to the drug law’s current draft legislation would introduce a more human rights-based approach, such as the abolition of prison time for first-time offenders, which would encourage participants to treat people with substance use disorders.

##### Social context

According to PCPs, the most stigmatized mental health conditions in Tunisia are substance use disorders, especially given the criminality (by law) associated with consumption and possession. However, PCPs mentioned that since the 2010–2011 Revolution, there has been a slow but steady shift in the community perception of people with substance use disorders:

*“Consumption' means that the person cannot control himself anymore. That's it, so we must consider him as a sick person and not as a social offender.” (Interview 5, participant 12)*

This perceptual change was instigated, according to PCPs, by increased drug circulation and consumption since the Revolution. PCPs mentioned that they also noticed anxiety and depressive disorders being less “taboo” in their practice than before the Revolution, since they are more common. This allows PCPs to “practice” what they learned during training.

Increased community awareness about mental illness, according to PCPs, is due to the Ministry’s prioritization of community-based mental health care. For example, the Ministry has recognized the need to decentralize mental health services by developing a *Committee for Mental Health Promotion* through which a mental health strategy was disseminated. Multiple initiatives have been undertaken to meet objectives listed in the strategy. First, PCPs mentioned that they noticed an increase in ways to help address negative attitudes towards mental illness:

*“I’ve noticed more television shows in the evening that invite many psychiatrists to talk about the recognition of cases of depression in Tunisia.” (Interview 7, participant 15)*

Secondly, the Ministry has been recently encouraging PCPs to record mental health statistics per primary healthcare clinic. Simply keeping statistics has increased participants’ awareness of mental illness in their practice. Lastly, PCPs believe the Ministry’s tactic to promote community-based mental health services is a way to counter the stigma of receiving care at the only operating mental health hospital in the country, *Razi Hospital*. Patients associate the hospital with alienation and a “place for the mad.”

While PCPs acknowledge decision-makers have a new interest in promoting mental health, challenges are still apparent. For example, PCPs are convinced that, compared to physical illness, mental illness is “forsaken”:

*“For hypertension and diabetes, there is an entire organization that deals with them. Statistics, drugs, care in general, people responsible for them are very thorough for these problems, which are international public health problems. But, for mental health […] mental health is not as well supported in the end.” (Interview 1, participant 1)*

Given this favoritism, PCPs noticed that decision-makers and clinic administrators are less concerned with “precise” mental health statistics than statistics for physical illnesses. In addition, the government documents on mental health and illness that PCPs consult are often outdated; they are rarely as frequently updated or distributed as those for physical illnesses.

##### Local workforce

PCPs shared current activities organized to develop the local workforce’s mental health capacities. First, given PCPs’ strategic position in the community and healthcare system, in 2011, the Ministry revamped the medical curriculum for future family physicians. It now includes additional mental health courses and a mandatory 2-month internship in post-graduate medical curricula for family physicians, previously optional. Therefore, under this medical education reform, all newly trained family physicians will be equipped with increased mental health care abilities.^3^ While participants shared approval for these much-needed additions to the medical school curriculum, they worried those untouched by the new mental health curriculum would be forgotten. Participants were quick to share their concern that the *mhGAP-IG* training would not be used to help fill gaps in competencies among newly graduated physicians and those untrained by the new curriculum. This apprehension emerged because continuity in mental health trainings rarely occurs:

*“Every time we do a mental health training program in Tunisia, a program where everybody is trying hard, everyone wants to be in this program, and after 2 or 3 months, 4 months, 5 months, there is no follow-up, no continuity, none.” (Interview 3, participant 9)*

Participants stated that if these mental health trainings based on the *mhGAP-IG* were to continue, not all PCPs could attend, preventing desired results from the intervention. They explained that in areas where physicians are scarce, not all can be excused from clinical duties to attend the training. This creates inconsistencies in mental health competency levels within and across regions. In addition, participants would have liked a mental health internship to complement the *mhGAP-IG* training. They believed this lacuna would also cause inconsistencies in mental health care, this time among current PCPs and recent medical school graduates under the new curriculum.

##### Aspects of the physical environment

PCPs shared that patients are inevitably referred to *Razi Hospital* given: 1) restrictions in place preventing physicians from prescribing certain medications listed in the *mhGAP-IG*; and 2) their perceived limited capabilities in addressing certain mental health conditions. Patients, however, are quick to refuse referrals to *Razi Hospital*, since it is far for most of them, public transportation to the hospital is limited, and taxi costs are high. In addition, consultation at *Razi* often requires long hours. A PCP explained that people living with psychosis are commonly required to travel up to 4 h to and from *Razi* and wait up to 2 h to see the psychiatrist. These barriers often instigate missed appointments, relapse, or, for patients who, on the rare occasion, may have the financial means, a push towards the private sector. Given prescription restrictions and their uneasiness with certain treatments even after training, PCPs feel like they cannot accommodate patients who miss appointments with their treating psychiatrists.

#### Organizational factors

PCPs highlighted more barriers (10 codes) than facilitators (4 codes) when describing organizational factors interacting with the implemented training to influence its expected outcomes.

##### Logistical issues

PCPs shared contrasting views on medication within their respective healthcare organizations. Some were satisfied with the types and amounts of medication available, but most mentioned they found it difficult to use the implemented *mhGAP*-based training, since no treatments beyond antidepressants were available. Participants added that if medications were available in primary healthcare clinics, they would often run out within days, which forces a “first come, first serve” philosophy. Given this philosophy and most patients’ inability to pay out-of-pocket for medication via the private sector, PCPs often noticed some patients remaining without medication for days. In addition, participants mentioned that if psychotropics were available in certain clinics, they could become targets of theft, given the drugs’ high street value since the 2010–2011 Revolution, and an increase in dependency related to their use.^4^ According to PCPs, theft poses severe security issues toward themselves, other healthcare personnel, and consulting patients.

Participants shared that even though they might have the will, knowledge, skills, and access to medication to address mental illness in practice, they cannot find the time to do so. Given their restricted work schedule (i.e., 8 h–14 h, Monday to Saturday) and the high patient volume (i.e., often over 25 patients per day), they feel as though they cannot adequately engage with people consulting for mental health issues. This affects their ability to offer adequate support.

PCPs shared two additional logistical barriers influencing the implemented training’s expected outcomes. First, participants working in peripheral regions of the Greater Tunis area said they often rotate primary healthcare clinics, which affects continuity in care. Patients who consult for mental health-related issues and return for further consultation might not be able to see the same doctor, making therapeutic alliance more difficult. Second, many participants worry about the high PCP turnover in primary healthcare clinics. As PCPs mentioned, more experienced PCPs usually practice in the Greater Tunis area, since younger doctors are solicited in Tunisia’s remote regions. Therefore, clinics in the Greater Tunis area often experience a high turnover of physicians; many leave for retirement or are solicited into administrative positions, which require quick replacement. High turnover affects the sustainability of mental health knowledge acquired through training within respective clinics.

##### Organizational culture: intra- and inter-collaboration

The *mhGAP-IG* training encourages collaboration with various healthcare professionals for cases requiring more expertise, or when specific issues challenge trainees. The training suggests specialists (i.e., psychiatrists, in the case of the Greater Tunis area) should be the “go to” for support. However, participants noted that since referral is done by letter, collaborations are difficult with the mental health hospital, where most psychiatrists work. To compensate for this barrier, participants said that within each governorate, physicians with more mental health knowledge and skills than the average PCP are available. Contacting these physicians is faster and easier than attempting to engage with specialists. Participants could rely on them during and after training if treatment questions arose. In addition, some PCPs mentioned they were fortunate to work near the few psychologists and social workers in the area. They would contact them if physicians with more mental health knowledge and skills were unavailable.

Participants recognized the importance of working with colleagues within their respective healthcare organizations to reinforce their knowledge and skills. While some PCPs stated they engage in monthly staff meetings where they discuss challenging mental health cases, most did not have this “luxury.” In addition, because the training was solely offered to PCPs, they often felt unsupported by other healthcare professionals at the primary healthcare clinic (i.e., nurses and paramedics), given their limited knowledge about the topic. For example, many participants mentioned nurses commonly questioned PCPs’ authority to provide mental health treatment or heard untrained medical staff using inappropriate, stigmatizing terms to refer to mental health patients. Thus, making mental health a priority within the primary healthcare organization was difficult post-training, given other healthcare professionals’ limited support and understanding.

#### Provider factors

PCPs highlighted more facilitators (6 codes) than barriers (4 codes) when describing provider factors interacting with the implemented training to influence its expected outcomes.

##### Providers’ previous medical experience

While most PCPs said the *mhGAP*-based training was the first they had ever attended, some did acknowledge previous participation in mental health training sessions dating back to the mid-2000s. Some trainings were provided by pharmaceutical representatives, who are well-versed on drugs to treat mental health problems, others were organized by representatives of governorates, consisting of theoretical sessions on bipolar disorder, depression, psychosis, schizophrenia, and treatment for substance use disorders. Few PCPs shared that they had chosen mental health internships during medical school. Regardless of participants’ previous experience, they all recognized the need to learn and/or refine mental health skills through the *mhGAP*-based training.

Interestingly, participants shared one commonality: certainty that their seniority as a PCP equipped them with superior general clinical abilities. Therefore, regardless of having participated in previous mental health training sessions or internships, PCPs felt pride in their ability to develop rapport with patients and engage in active listening, skills they thought helped them better assimilate general principles of care for people living with mental health problems:

*“Consultation with chronic patients is an individualized practice. So, the attending physician is the doctor in which the patient confides, even independently of mental health problems. In mental health, there is the same listening. That is, we have practiced it in other areas, other than mental health.” (Interview 8, participant 16)*

##### Providers’ personal characteristics

According to participants, personal interest led to their participation in the *mhGAP*-based training. This is alluded to in how the training was provided on a voluntary basis outside of office hours. Most PCPs said they attended the training because they had developed personal preferences for certain types of mental health conditions (i.e., depression) and they knew the training would highlight them.

It is also important to note, however, that even though interviewees participated in the *mhGAP*-based training, some of their views may have challenged the implemented program’s expected outcomes. Firstly, some PCPs were still not enthralled by mental health care after training but forced themselves to engage with people presenting with mental health conditions given their rise in frequency. Hence, practicing mental health was an effort for them, some even calling it “unpleasant.” Secondly, PCPs mentioned that despite the training, they did not feel comfortable treating certain types of mental health conditions and never would. These include psychosis and substance use disorders. Lastly, some PCPs did not understand their role in prescribing medication to treat mental illness. They believed it was beyond their capacities, even with training, and therefore they have no interest in this form of treatment.

#### Patient factors

PCPs highlighted more barriers (7 codes) than facilitators (8 codes) when describing patient factors interacting with the implemented training to influence its expected outcomes.

##### Patients’ beliefs about the health system and its professionals

According to participants, patients prefer avoiding *Razi Hospital* for mental health care. The hospital’s stigma makes them believe that if referred there, it is because they are “crazy,” “unrecoverable,” and “deranged.” Patients are also less likely to seek care at the hospital because it is far for most and requires an entire day to be treated, given high demand for specialists. Therefore, PCPs believe patients will be more inclined to seek mental health care at the primary healthcare clinic. The primary healthcare clinic is less stigmatizing, and patients’ issues may be difficult for others to identify amid the vast range of consultations:

*“When people with mental health conditions receive care within primary care clinics, they will be integrated with the common person, that is to say no one will know if consultation will be for depression, an angina, or for other reasons. That's the positive side.” (Interview 8, participant 16)*

However, some PCPs worried that patients might not readily seek mental health care within clinics because, until recently, mental health care has been primarily encouraged within institutions. In addition, patients know that the prescription of certain treatments, given restrictions, are solely reserved for psychiatrists. Therefore, some patients might be wary that mental health services offered by trained non-specialists are not as effective as specialists’ care.

##### Patients’ motivation to seek care

Participants highlighted multiple barriers to patients’ motivation to seek care. Despite a noticeable push to raise mental illness awareness, participants noticed most patients prefer avoiding mental health consultations. Patients are therefore “forced” to consult by worried family members or friends. Participants identified two reasons for this demotivation. First, patients fear other consulting community members recognizing them at the primary healthcare clinic, most of whom know each other. Being recognized is problematic especially in the case of substance use disorders, given the legal repercussions of consumption and possession. In addition, the fear of being treated differently leads to patients’ demotivation to seek care. For example, PCPs noticed that patients officially diagnosed with a mental health condition often lose trustworthiness and are labelled “deviant”:

*“Having a mental illness means we do not trust you anymore, it means that we are afraid of you, it means […] we're not going to give you money because you're going to lose it. You're not doing well, you are not normal, you are pathological. I cannot give you the keys of my car. His mom, his dad, his brother, his friend, they will not trust him anymore.” (Interview 1, participant 1)*

Logistical issues also influence motivation to seek care. According to participants, because the *mhGAP*-based training was a pilot initiative in the Greater Tunis area, most patients are not aware some PCPs have participated in the program and can provide effective mental health care. If, by chance, patients are aware PCPs have been newly trained, they worry that services are not confidential. For example, patients were wary of providing a reason for consultation to the welcome staff (i.e., secretariat) at the clinic out of fear that this might be shared with others and thus increase their chances of being labelled negatively by other community members. Lastly, participants shared that the interruption of patients by others waiting to be seen by physicians is common in Tunisia, which makes patients uncomfortable, especially when consulting for mental health-related issues.

Encouragingly, participants shared a logistical issue they believe would promote the use of their competencies and skills acquired through the *mhGAP*-based training. Most patients will inevitably seek care at the primary healthcare clinic between scheduled appointments with psychiatrists if complications occur. Therefore, given specialists’ unavailability beyond scheduled appointments, PCPs may be used as “fillers” between appointments, if they feel capable of addressing the mental health concern. Satisfied with services received through this type of unexpected consultation, some patients have even asked to be transferred to PCPs’ care.

#### Innovation factors

PCPs highlighted more facilitators (7 codes) than barriers (5 codes) when describing characteristics of the training program that facilitated or challenged the attainment of its expected outcomes.

##### Program’s compatibility with clinical practice

Participants shared that their perception of the implemented training’s clinical utility influenced the intervention’s ability to ensure the attainment of desired outcomes. First, they shared that the modules chosen for the training program correspond to realities seen in their everyday practice. They confirmed that they see depression cases daily, while conditions related to other modules covered (i.e., psychosis, self-harm/suicide, and substance use disorders) are also seen. Second, PCPs shared that the modules were well-chosen because they consider the Greater Tunis area’s mental health trends, especially since the Revolution. However, PCPs cautioned against excluding what they considered clinically useful modules. Since PCPs conduct clinical practice in schools weekly, they were surprised that modules on developmental and behavioural disorders were omitted, and that there was little to no information on youth mental health topics. In addition, given limited dementia and epilepsy specialists, PCPs said they need training for these disorders, which was also omitted.

##### Program’s quality

PCPs evaluated the degree of the program’s quality based on its practicality. For example, since many PCPs rarely discuss clinical cases with colleagues in their respective healthcare organizations, they appreciated the time allocated for clinical discussion during training sessions. These discussions, as shared by participants, helped orient future practice, and provided the opportunity for peer learning. In addition, PCPs enjoyed role plays, especially since this facet of training was novel to them. According to participants, role plays helped orient their questions about mental illness to facilitate detection and better their general approach with patients. However, participants thought that the implemented training program overly focused on theory, a reality even acknowledged by PCPs who did not have previous mental health experience. Importantly, participants thought practicality would aid them much more than theory, given their confidence in general clinical skills acquired through years of experience:

*“I would have liked something more practical because at our age and with our experience attending a theoretical class is not very interesting. What we have in the handout is very clear. All they [the trainers] did was re-read it for the general lecture. So, it was not very practical.” (Interview 7, participant 15)*

Participants also thought that the degree of the training program’s quality was related to the type of mediums presented to them. Such mediums, they highlighted, helped them better assimilate the training program’s content. Specifically, PCPs appreciated the videos, as they illustrated effective clinical mental health encounters between healthcare workers and patients. Participants who had participated in previous mental health training programs mentioned that they had never seen videos illustrating effective mental health practice with patients. In addition, PCPs appreciated receiving the *mhGAP-IG* manual because they were accustomed to consulting outdated mental health pamphlets, if any at all. The guide’s practicality empowered PCPs during and after training because they felt that knowledge was “at their fingertips.” Beyond practicality, knowing that the guide was created by the *WHO*, and that the training was supported by members of the Ministry of Health and the *WHO* office in Tunisia, PCPs felt as though they were included in a global movement for better mental health care.

PCPs also mentioned barriers to attaining the implemented training’s expected outcomes. Firstly, the guide (i.e., the *mhGAP-IG version 1.0*) provided to all trainees contains thirteen modules. PCPs questioned why they were only taught six modules, especially since training resources were already mobilized. Secondly, PCPs questioned the training schedule. The training was offered after their clinical practice, one afternoon a week for 6 weeks. In this short time, they thought too much content was provided, which influenced some of their colleagues’ decisions to drop out of the program. Participants would have preferred training over the entire day, with theoretical sessions in the morning and the rest of the day reserved for more practical aspects (i.e., role plays, small group discussions, and clinical case presentations). Lastly, some PCPs, while a minority, were displeased with the role plays. They felt uncomfortable, “put on the spot,” and nervous. During role plays, PCPs were often asked to role play as patients, which they found difficult. They thus believed that their inability to adequately represent a consulting patient jeopardized the goal of the role plays: to reinforce theoretical learning through practice.

### Part 3: Potential solutions suggested by trained PCPs

Participants offered recommendations to address contextual factors they believe interacted with the implemented training to influence its expected outcomes (i.e., desired competencies and skills). These recommendations are useful given that they derive from trainees with in-depth understanding of the components of the implemented training and the factors within their immediate and broader environment that interacted with the program to influence its expected outcomes.

#### Improving the broader context

To ensure expected outcomes are attained by the implemented training program, PCPs suggested further considering the standardization of mental health practice. For example, PCPs mentioned the necessity of ensuring that mental health resources, such as psychiatrists, psychologists, social workers, and medications, all listed in the *mhGAP-IG*, are equitably distributed across the country. To ensure resources meet current mental health needs, PCPs suggested that decision-makers pay better attention to gaps in mental healthcare delivery, particularly by inquiring about primary care realities experienced across the country and visiting areas where the *mhGAP*-based training will be offered.

According to PCPs, the standardization of mental health care delivery to help reach the implemented training’s desired outcomes also means providing practical solutions to encourage PCPs’ roles in mental health care. Interestingly, these suggestions mirror the current practice for other chronic illnesses, such as diabetes and hypertension. Participants shared the utility of dedicating a person responsible for mental health within each governorate. This person would be in contact with PCPs to inquire about current mental health statistics and encourage evidence-based practice, examples of which are listed in the *mhGAP-IG*. In addition, PCPs saw the advantage of encouraging appointment scheduling for people consulting for mental illness, which would allow them more time in consultation and facilitate continuity in services.

Consensus among PCPs is that in Tunisia, mental health training programs are initiated, but rarely sustained, a reality that may prevent the sustainability of the implemented training’s desired outcomes. Hence, training programs and refresher courses for PCPs should be prioritized. Participants also suggested mental health internships in continuing medical education should be offered to integrate knowledge, since PCPs are legally entitled to excuse themselves from clinical practice to pursue practical learning in any discipline.

In addition, PCPs confirmed that support from and collaboration with specialists is essential to reinforce the competencies and skills developed through training. First, specialists’ help with challenging cases is viewed as vital, especially when side effects from medications are apparent. PCPs lacked this knowledge even after training. Second, participants said their new competencies and skills may be furthered by encouraging a culture of retroactive feedback. PCPs expressed the need for specialist feedback on cases they refer. This lack of feedback is detrimental to the training’s application and affects continuity in care.

While these listed recommendations are imperative, they become ineffective if PCPs continue to have restrictions regarding the prescription of certain medications suggested by the training guide.

#### Improving the organizational context

Participants listed logistical challenges within healthcare organizations that they thought interacted with the implemented training to challenge its expected outcomes. They provided recommendations to address one of these challenges. Participants hoped their organizations would encourage mental health discussions among colleagues. They suggested having someone within the organization, such as a PCP or an administrator, organize time for such discussions, where challenging cases and queries about medication may be presented. Participants believe this space for mental health dialogue could ensure mental health’s prioritization in practice and further encourage collaboration within the organization.

#### Improving the mental health training program

Participants suggested ways to improve the training program, which, according to them, might help better achieve its desired outcomes. Firstly, all participants suggested making the program more practical. Specifically, they suggested: facilitating additional clinical case discussions beyond the 2-h session provided; including a mandatory internship after the training to complement theoretical learning; providing substantially more information on conducts for mental health treatment; including more role plays to further facilitate knowledge integration; and providing PCPs with clinical tools to ensure they can adequately pose a mental health diagnosis in consultation. While participants appreciated the guide and its accompanying master chart highlighting the common presentations of priority conditions to be assessed, they would also like specific tools such as questionnaires with suggested cut-off scores to help concretely diagnose patients.

Secondly, all participants said future trainings should better reflect contextual realities experienced in primary healthcare clinics so as to be more clinically useful. For example, PCPs suggested: 1) including more information on treatments for substance use disorders and general pharmacology, specifically with regards to side effects and interactions between medications; 2) providing information on therapy with patients, specifically cognitive-behavioral therapy, given limited availability for such training in Tunisia [56]; and 3) prioritizing modules pertaining to youth mental health, to facilitate their responsibilities in schools.

Lastly, participants suggested ways to address the logistical issues of the implemented training program, which they believed prevented the attainment of its desired outcomes. PCPs did not appreciate being “rushed” to learn about mental health over a brief period (6 weeks). Thus, participants suggested elongating the training and adding more sessions to cover additional topics. In addition, PCPs suggested finding an alternative schedule. Participating in the training in the afternoon after a day of consultations, as was done, made it hard to retain information. Furthermore, while PCPs were provided with a pamphlet regrouping copies of the presentation slides, they thought this redundant information. For the next trainings, they suggested documents be written succinctly, with easy take-home messages from the theoretical presentations, group discussions, and role plays.

## Discussion

This paper provides a glimpse into the complexity of offering a mental health training based on the *mhGAP-IG* to PCPs working in the Greater Tunis area of Tunisia given contextual factors that interacted with the implemented intervention to influence its expected outcomes. Results from this Type III implementation analysis [33] are useful for two main reasons. First, findings may inform results obtained on mental health knowledge, attitudes, self-efficacy, and self-reported practice questionnaires from our randomized controlled trial [43]. For example, in this paper, we presented more barriers (37 codes) than facilitators (31 codes) when identifying contextual factors influencing the implemented training’s desired outcomes. PCPs still felt uncomfortable with certain aspects of treatment despite their participation in the training program, specifically in pharmacology and with specific mental health conditions, such as psychosis, schizophrenia, and substance use disorders. Therefore, we expect to find lower scores for these criteria on the questionnaires.

Second, at the heart of this paper is Tunisia’s interest in building non-specialists mental health capacities, which is also an international effort to further develop effective mental health services in primary care settings [4, 23, 25]. Therefore, in addition to informing our randomized controlled trial, our findings uncovered contextual factors that can be tailored to prepare for future implementations of the *mhGAP*-based training in Tunisia’s other regions and address the mental health treatment gap [41, 43, 56]. Decision-makers may rely upon participants’ in-depth knowledge about their communities and primary healthcare organizations to improve the training program and environment in which it was (and will be) implemented [58]. Such findings also contribute to a research priority in global mental health: generating evidence on communal factors supporting the involvement of non-specialists’ in mental health care delivery [67]. This evidence may be used as a guide to improve health services in LMICs while being sensitive to local particularities [67–69].

As suggested by authors who have engaged in developing non-specialists’ mental health capacity through offering training programs: *“making it easier for generalists to acquire and practice skills in the recognition of and treatment of mental health problems […] is not sufficient, and it will not be possible to meet need by continuing to pursue the idea of simply training more people”* [67]. Therefore, to optimize PCPs’ role in the field of mental health in Tunisia, initiatives beyond training are fundamental. These include modifications to structural and organizational factors [35]. Interestingly, previous studies have observed key structural and organizational challenges facing non-specialists’ provision of mental health care in LMICs that are similar to the ones we have identified [37, 68, 69]. Similar barriers include: 1) challenging policies (in our case, restrictions preventing PCPs from prescribing certain medications and the criminalization of substance use disorders); 2) mental health training (in our case, lack of continuity in mental health trainings and limited encouragement for participation in mental health internships, part of continuing medical education); 3) mental health resources (in our case, limited availability and uneven distribution of medications); and 4) organization and planning (in our case, obstacles to continuity in care, lack of time to provide mental health care, high turnover of trained employees, other professionals’ limited support for the integration of mental health into primary care, and limited mental health support).

Two aspects of our findings surprised us. First, participants did not allude to a structural factor that authors have previously identified when reviewing the feasibility and acceptability of relying on non-specialists for mental health care in LMICs: funding allocated to mental health [68]. While mental health funding may be beyond the scope of PCPs’ comprehension, it nonetheless remains an important structural factor to consider when aiming to decentralize mental health services by further relying on primary care settings and the involvement of non-specialists in mental health care delivery [4, 10, 16, 24]. With limited government investment allocated to mental health in LMICs, Tunisia included, most funding continues to sustain institutional settings [16, 24, 70]. Focusing on institutional settings thus poses a severe threat to future trainings based on the *mhGAP-IG* [4, 23–25] and to the use and sustainability of competencies and skills acquired through training [68, 69].

Another surprising aspect of our findings pertains to a comparison between our results and those by Chaudoir and colleagues (2013) [57], who state in a review that they were least likely to come across variables related to structural and patient factors. Interestingly, when exploring contextual factors interacting with the implemented training program to influence its desired outcomes, our findings show that the study’s participants were primarily concerned with these two types of constructs. Structural factors (e.g., policies, social context, development of the local workforce, and physical aspects of the environment) and patient factors (e.g., beliefs about the health system and healthcare professionals, as well as motivation to seek care) were addressed by more codes than organizational, innovation, and provider factors alone. We explain the discrepancy between Chaudoir and colleagues’ (2013) findings [57] and ours in several ways. First, the use of non-specialists in mental health care delivery at the level of primary care generates a new vision countering the long-standing position of institutional-based mental health care in LMICs. This new vision upholds the key features of primary care services outlined by Starfield [71], such as first-contact, comprehensive, and coordinated care. Thus, relying on trained non-specialists inevitably requires a re-structuring of systemic and organizational factors in order to create and support a healthcare system ready to welcome new treatment and management roles. These roles include non-specialists’ increased involvement in detection, treatment, and management, with the role of specialists consisting of consultation, supervision, and further trainings [12, 13]. However, despite the Ministry’s prioritization of mental health in Tunisia, our findings highlight significant barriers that may challenge these new roles. These include: restrictions limiting PCPs’ prescribing power, the questioning of mental health care in primary care settings, and deficits in continuing (and sustained) medical education programs targeting mental health.

Second, as participants shared, patients prefer seeking mental health care at local primary healthcare clinics rather than at institutions, which suggests patients’ approval of offering mental health training to non-specialists such as PCPs. However, according to PCPs, patients are still affected by sociocultural nuances (i.e., the perception of mental health and mental health care) within the broader context, which PCPs believe influence their help-seeking behavior even within primary healthcare clinics. For example, our study’s participants suggest patients are wary of trained PCPs because they are not “specialists.” In addition, the stigma against mental illness worries patients. For example, patients fear being treated differently if they are labeled with a mental health condition. As other studies suggest, positive effects resulting from targeting such sociocultural nuances within the broader context may trickle down to the micro level to improve patients’ willingness to seek help confidently within the community [68, 69, 72].

### Limitations

Limits to the study should be noted. Firstly, our sample consists of PCPs working in the public sector from one area of Tunisia. Implementing the training in different areas of Tunisia and interviewing participating PCPs from those areas could result in additional contextual factors interacting with the program to influence its expected outcomes. Nonetheless, we believe our findings are quite comprehensive and useful because PCPs in the Greater Tunis area experience similar barriers to effective mental health care as in other regions. Secondly, we captured participants’ perceptions of barriers and facilitators interacting with the implemented training to influence its expected outcomes at one time, shortly after the intervention’s completion. While this short-term follow-up is valuable, long-term follow-up could inform decision-makers how contextual factors interacted with the implemented training program to influence the evolution of desired outcomes. Thirdly, the training’s expected outcomes, as listed in this paper in Part 1 of the results section, are based on participants’ perceptions. While this information is useful to complement our randomized controlled trial, results obtained on mental health knowledge, attitudes, perceived self-efficacy, and self-reported practice questionnaires from the trial might better reflect the acquired competencies and skills from the implemented training. In addition, participants shared what they believed impacted patients’ help-seeking behaviour. Interviewing people with mental health problems who consulted trained PCPs would thus have been useful to confirm or complement these perceptions. Lastly, this paper presents contextual factors interacting with the implemented training to influence the training’s expected outcomes (i.e., a Type III implementation analysis). In retrospect, exploring how contextual factors impacted the planned implementation of the training program would have been beneficial (i.e., a Type I implementation analysis) [33]. This complementary information might have painted a more accurate picture of the implemented program and its interaction with contextual factors in the context of the Greater Tunis area.

## Conclusion

This case study highlights the complexity of implementing a *mhGAP*-based training in the Greater Tunis area of Tunisia given its interaction with contextual factors to hinder or facilitate the attainment of its expected outcomes. While participants did acknowledge the implemented training’s many positive effects on their competencies and skills, post-training, contextual barriers prevented them from feeling comfortable with certain aspects of treatment and the management of specific mental health conditions. Hence, in order to ensure PCPs’ effective involvement in mental health care, contextual barriers interacting with the implemented training as identified in this paper should be addressed before future implementations of a *mhGAP*-based training. Findings may also be used by decision-makers of other LMICs interested in implementing a *mhGAP* based training yet facing similar challenges in further involving non-specialists in effective mental health care delivery at the level of primary care.

## Additional files

Additional file 1: Examples of interview questions. (DOCX 20.5 kb)

Additional file 2: Final Code book. (DOCX 33.2 kb)

## Abbreviations

IG: Intervention guide
LMICs: Low- and middle-income countries
mhGAP: Mental Health Gap Action Programme
PCPs: Primary care physicians
WHO: World Health Organization

## Footnotes

1 Numbers in brackets highlight individuals from each governorate who participated in the group interviews.

2 A circular exists limiting the prescription of certain psychotropic drugs (ex.: benzodiazepines) at the level of primary care. This regulation preventing the prescription of these psychotropic drugs was not revised after the implementation of training initiatives targeting PCPs’ mental health competencies.

3 The first wave of family physicians who participated in this new medical curriculum is scheduled to graduate in 2019.

4 In certain healthcare clinics, psychotropic drugs have been subject to theft, as they are often coveted in the Greater Tunis area by people living with substance use disorders. Measures have been taken to limit the availability of psychotropic drugs in healthcare clinics where reports of theft have been made. Consequently, these measures create uneven distribution of such drugs in primary care settings.
