# Supplementary material for: “We find what we look for, and we look for what we know”: factors interacting with a mental health training program to influence its expected outcomes in Tunisia
Source: BMC Public Health. 2018 Dec 20;18:1398. doi: 10.1186/s12889-018-6261-4 (PMC6302293; doi:10.1186/s12889-018-6261-4)
Supplement: Supplementary file 1 — Examples of interview questions. (DOCX 20.5 kb) [file 12889_2018_6261_MOESM1_ESM.docx]

“We find what we look for and we look for what we know”: factors interacting with a mental health training program to influence its expected outcomes in Tunisia (Spagnolo et al. (2018))

“Additional File 1” – Examples of interview questions

**Example of Interview Questions**

**Theme 1: Structural factors**

Why was a mental health training program offered to primary care physicians in the Greater Tunis area of Tunisia? (N.B.: Probe: who normally organizes mental health trainings and at what frequency, etc.)

How does the political or social climate in the Greater Tunis area facilitate or hinder the use or outcomes (i.e., mental health knowledge, attitudes, self-efficacy, and practice) of the implemented training program?

How do public policies facilitate or hinder the use or outcomes (i.e., mental health knowledge, attitudes, self-efficacy, and practice) of the implemented the training program?

How do aspects of the physical environment in the Greater Tunis area facilitate or hinder the use or outcomes (i.e., mental health knowledge, attitudes, self-efficacy, and practice) of the implemented the training program?

**Theme 2: Organizational factors**

How are mental health services organized in your governorate/delegation? What is the effect of this organization on your mental health care delivery?

Given the organization of mental health services within your governorate/delegation, what organizational factors facilitate or hinder the use or outcomes (mental health knowledge, attitudes, self-efficacy, and practice) of the implemented the training program?

What factors within your specific healthcare organization facilitate or hinder the use or outcomes (i.e., mental health knowledge, attitudes, self-efficacy, and practice) of the implemented the training program? (ex: culture of learning, supervisors/administrators, etc.)

**Theme 3: Provider factors**

Why did you sign up for the mental health training?

What experience (i.e., participation in previous training programs, internships, courses, etc.) do you have in mental health?

What aspects of the implemented training program were the most important to your clinical practice?

What was the impact of the training program on your clinical practice? What impact of the training program on your clinical practice did you hope to see, but, unfortunately, did not?

**Theme 4: Patient factors**

What types of mental health consultations do you receive in your clinical practice?

What impact has the implemented training program had on your patients consulting for mental health problems?

What changes (positive or negative) have you seen in your patients consulting for mental health problems after you participated in the training?

**Theme 5: Innovative factors**

What is your opinion about the structure (i.e., a session once a week, theory and practice, support session at the end of the training program, trainers and tutors, etc.) of the training program?

As a trainee, what did you like about participating in the training program?

As a trainee, what did you dislike about participating in the training program?

In your opinion, is there anything missing from the training program that you would have found useful?

What aspects learned in the training program do you plan to use in clinical practice?

How does this mental health training program compare with others you have received?
