# Supplementary material for: “We find what we look for, and we look for what we know”: factors interacting with a mental health training program to influence its expected outcomes in Tunisia
Source: BMC Public Health. 2018 Dec 20;18:1398. doi: 10.1186/s12889-018-6261-4 (PMC6302293; doi:10.1186/s12889-018-6261-4)
Supplement: Supplementary file 2 — Final Code book. (DOCX 33.2 kb) [file 12889_2018_6261_MOESM2_ESM.docx]

“We find what we look for and we look for what we know”: factors interacting with a mental health training program to influence its expected outcomes in Tunisia (Spagnolo et al. (2018))

“Additional File 2” – Final code book

| ***Parts*** | ***Themes*** | ***Sub-themes*** | ***Codes*** |
| --- | --- | --- | --- |
| Part 1:  The effects of the training on primary care physicians’ (PCPs) competencies and skills. | 1. Describing the effects of the training on PCPs’ competencies. | 1a) PCPs’ knowledge about mental health and illness. | - (+) PCPs are more familiar with medication. - (+) PCPs are more knowledgeable about ways to approach mental illness in clinical practice. - (+) PCPs are more knowledgeable about symptoms related to mental illness. - (-) PCPs are still unfamiliar with medication. |
|  |  | 1b) PCPs’ attitudes towards mental health and illness. | - (+) PCPs acquired a better understanding of the “suffering” associated with mental illness. - (+) The training helped demystify the management of mental health issues in primary care settings. - (+) The training targeted the negative beliefs about certain mental health issues. - (+) PCPs allocate more time to mental health during practice. - (+) PCPs are more patient with people consulting for mental health issues. - (-) PCPs are still afraid of treating certain types of mental health conditions. |
|  | 2. Describing the effects of the training on PCPs’ practice. | 2a) PCPs’ detection skills to address mental health issues. | - (+) PCPs feel confident asking “good” questions to inquire about mental health and illness. - (+) PCPs are more inclined to check mental health in regular consultation. - (+) PCPs can more easily detect symptoms related to mental illness in patients. |
|  |  | 2b) PCPs’ treatment skills to address mental health issues. | - (+) PCPs are more inclined to consider psychosocial treatment. - (+) PCPs have more confidence to prescribe. - (+) PCPs are more confident to see (and treat) a greater number of patients with mental health problems. - (+) PCPs try to ensure a greater continuity in care. - (-) PCPs are not confident in providing treatment using certain types of medications. - (-) PCPs are not confident in treating certain types of mental health conditions. - (-) PCPs are not confident in managing and following-up on treatments for certain patients. |
| Part 2:  Contextual factors that interact with the implemented training program to influence its expected outcomes (illustrated in Part 1).  This part is organized according to Chaudoir and colleagues’ (2013) [57] conceptual framework. | **DIMENSION 1:**  **STRUCTURAL-LEVEL FACTORS** |  |  |
|  | 1a) Public policies (i.e., laws and restrictions) interact with the training program to influence its expected outcomes. | Mental health laws and restrictions affecting adequate mental health practice. | - (barrier) PCPs cannot prescribe certain molecules. - (barrier) Substance use disorders are often managed judicially. - (facilitator) Laws and restrictions are changing to reflect current trends in mental health. |
|  | 1b) The social context (perceptions, values) interacts with the training program to influence its expected outcomes. | The Ministry’s prioritization of mental health care in the country. | - (barrier) PCPs feel that physical health is valued more than mental health. - (barrier) Mental health statistics are not taken seriously. - (barrier) PCPs still use “ancient” mental health tools in practice. - (facilitator) There is an increased attention put on mental health statistics. - (facilitator) Mental health is recognized in the country through the development of the national programme for mental health promotion. - (facilitator) Strategies are used to increase awareness of mental health conditions across the country. |
|  |  | The perception of mental health conditions in Tunisia. | - (barrier) Substance use disorders are stigmatized in Tunisia. - (facilitator) There is less stigma towards certain types of mental disorders since the 2010-2011 Revolution. |
|  |  | The perception of mental health care within institutions | - (barrier) Mental health care within institutions is stigmatized by the community. |
|  | 1c) Infrastructure (i.e., the local workforce) interacts with the training program to influence its expected outcomes. | The development of non-specialists’ mental health capacities. | - (barrier) Lack of continuity in mental health trainings. - (barrier) Lack of obligatory mental health internships in continuing medical education to further develop professional practice. - (barrier) If there are mental health trainings, not all PCPs can attend. - (facilitator) The Ministry adopted a new medical curriculum, encouraging increased teachings and internships in mental health for future family physicians. |
|  | 1d) The physical environment (i.e., topographical elements that pose barriers or encourage clinical access) interacts with the training program to influence its expected outcomes. | Difficulty accessing the mental health hospital and its services. | - (barrier) There is only one mental health hospital in the country, and it is not accessible to all. |
|  | **DIMENSION 2:**  **ORGANIZATIONAL-LEVEL FACTORS** |  |  |
|  | 2a) The logistical issues within the healthcare organization interact with the training program to influence its expected outcomes. | There are logistical issues when providing mental health care within primary healthcare clinics. | - (barrier) Trained PCPs are not always at the same primary healthcare clinic, which affects continuity in care. - (barrier) There is a lack of medication in primary healthcare clinics. - (barrier) If medication is available, it is easily stolen in certain primary healthcare clinics. - (barrier) If medication is available, it is not evenly distributed. - (barrier) If medication is available, it runs out quickly. - (barrier) Lack of time to provide adequate mental health care. - (barrier) High turnover of employees within healthcare organizations. - (facilitator) Medication is available within primary healthcare clinics. |
|  | 2b) The organizational culture (i.e., a system of shared beliefs, values, and assumptions about care) interacts with the training program to influence its expected outcomes. | Intra-collaboration. | - (barrier) PCPs expressed difficulties working with other healthcare professionals in the primary healthcare clinic. - (barrier) Primary healthcare clinics do not promote staff meetings. - (facilitator) PCPs engage in case discussions with colleagues about mental health. |
|  |  | Inter-collaboration. | - (barrier) Collaborations with the mental health hospital is difficult. - (facilitator) Collaborations with PCPs responsible for continuing medication education helps with mental health care delivery. - (facilitator) There are opportunities for collaborations with other healthcare professionals. |
|  | **DIMENSION 3:**  **PROVIDER-LEVEL FACTORS** |  |  |
|  | 3a) Providers’ previous medical experiences interact with the training program to influence its expected outcomes. | PCPs’ involvement in mental health activities during their careers. | - (barrier) PCPs do not have previous mental health training. - (facilitator) PCPs participated in previous mental health trainings. - (facilitator) PCPs participated in a mental health internship during medical school. |
|  |  | Providers’ seniority in the field as a PCP. | - (facilitator) Many years of field experience have equipped PCPs with confidence in their general clinical skills. |
|  | 3b) Providers’ personal characteristics interact with the training program to influence its expected outcomes. | PCPs’ desire to learn about and provide mental health care stems from personal interest. | - (barrier) PCPs do not like treating certain types of mental health conditions. - (barrier) PCPs do not get involved with pharmacological treatment. - (barrier) PCPs are not interested in mental health. - (facilitator) PCPs are personally motivated to provide mental health care. - (facilitator) PCPs have personal preferences for certain types of mental health conditions. - (facilitator) PCPs participate in mental health training during their own time (outside of office hours). |
|  | **DIMENSION 4:**  **PATIENT-LEVEL FACTORS** |  |  |
|  | 4a) Patients’ beliefs about the health system and its professionals interact with the training program to influence its expected outcomes. | Help-seeking behaviour is influenced by perceptions of primary healthcare clinics. | - (barrier) Patients think that receiving care in primary healthcare clinics is sub-par to receiving care by a specialist. - (facilitator) Patients prefer seeking and receiving care at the primary healthcare clinic because it is less stigmatizing than the mental health hospital. - (facilitator) Patients like receiving care at the primary healthcare clinic because they are not noticed. - (facilitator) Patients like receiving care at the primary healthcare clinic because it is offered quickly. |
|  |  | Help-seeking behaviour is influenced by perceptions of the mental health hospital. | - (facilitator) Patients think that the mental health hospital is very stigmatizing. - (facilitator) Patients think that the mental health hospital is too far. - (facilitator) Patients think that receiving services at the mental health hospital takes too long. |
|  | 4b) Patients’ motivation to seek care interacts with the training program to influence its expected outcomes. | Motivation to seek care is influenced by views of mental illness. | - (barrier) Patients are treated differently once “society” knows they live with mental health issues. - (barrier) Patients do not seek care because they are afraid of legal issues. - (barrier) Patients do not seek care because they do not want to be noticed by community members. - (facilitator) Patients are more open about their own mental health. |
|  |  | Motivation to seek care is influenced by logistical issues in primary healthcare clinics. | - (barrier) In consultation, patients consulting for mental health conditions are interrupted by other patients. - (barrier) Patients are not aware that mental health services are available at the primary healthcare clinics. - (barrier) Patients do not know that mental health services are confidential. - (facilitator) Patients will seek care at the primary healthcare clinic between appointments with psychiatrists. |
|  | **DIMENSION 5:**  **INNOVATION-LEVEL FACTORS** |  |  |
|  | 5a) PCPs’ perception of the training’s compatibility with the context in which it was implemented is a factor that influences its expected outcomes. | Training modules were clinically useful. | - (barrier) Training modules chosen do not correspond to the clientele seen by PCPs. - (facilitator) Training modules chosen correspond to the reality seen by PCPs. - (facilitator) Training modules chosen correspond to the reality of the Greater Tunis area. |
|  | 5b) PCPs’ perception of the program’s quality is a factor that influences its expected outcomes. | PCPs’ perception of the program’s practicality. | - (barrier) PCPs did not like all the theory provided during the training. - (facilitator) PCPs appreciated the clinical discussions during the training as they helped orient future practice. - (facilitator) PCPs liked the role plays because they helped learning. - (facilitator) PCPs liked that they could learn from their peers. |
|  |  | PCPs’ perceptions on training content. | - (barrier) PCPs did not like that they were not able to learn about all the modules included in the training guide. - (barrier) PCPs did not like role plays. - (barrier) PCPs found there was not enough time for all the content provided. - (facilitator) PCPs enjoyed the videos shown during the training. - (facilitator) PCPs liked the training guide. |
| Part 3:  Potential solutions to address contextual factors, as suggested by trained PCPs | 1. Improving the broader context in which a mental health training program is implemented, to influences its expected outcomes. | 1a) Further developing national capacity for mental health care. | - Availability of sufficient mental health resources (psychiatrists, psychologists, social workers, medication) across the country. - Assigning a person responsible for mental health within each governorate. - Informing the community that mental health services are available through trained PCPs. - Constantly evaluating mental health care and resources. |
|  |  | 1b) Building PCPs’ capacities in mental health. | - Ensuring continuity of mental health training programs. - PCPs would like support from psychiatrists when working with challenging cases. - PCPs would like feedback on their referrals to specialized services. |
|  |  | 1c) Ensuring that PCPs do not have restrictions for the prescription of needed medications. | - Removing restrictions that prevent PCPs from prescribing certain types of medications. |
|  | 2. Improving the organizational context in which a mental health training program is implemented, to influence its expected outcomes. | 2b) Developing a greater culture of learning within the organization. | - Having someone within the primary healthcare clinic organize discussions on mental health among colleagues. |
|  | 3. Improving the mental health training program, to influence its expected outcomes. | 3a) Making the training more practical. | - Encouraging more clinical case discussions. - Including an internship after the training program. - Focusing more on “what to do” (*conduite à tenir*) for people presenting with mental health related issues in primary healthcare clinics. - Providing PCPs with clinical tools (ex.: questionnaires) to help diagnose and treat. |
|  |  | 3b) Making the training more clinically useful. | - Need for more information on therapeutic endeavors with patients. - Need for more training on substance use disorders. - Need for more training modules (i.e. youth mental health, epilepsy, dementia). - Need for more training on pharmacology. - Need for additional role plays. |
|  |  | 3c) Addressing the logistical issues of the training. | - Adding more sessions and topics. - Providing various kinds of materials to participants, in complement to the guide. - Altering the schedule of the training. |
